# Effect of changes in cerebral oximeter values during cardiac surgery on the incidence of postoperative neurocognitive deficits (POND): A retrospective study based on propensity score–matched analysis

**DOI:** 10.1371/journal.pone.0260945

**Published:** 2021-12-03

**Authors:** Jin Hee Ahn, Eun kyung Lee, Doyeon Kim, SeHee Kang, Won-Jun Choi, Jae-hun Byun, Jae-Geum Shim, Sung Hyun Lee

**Affiliations:** 1 Department of Anaesthesiology and Pain Medicine, Kangbuk Samsung Hospital, Sungkyunkwan University School of Medicine, Seoul, Korea; 2 Department of Anaesthesiology and Pain Medicine, Samsung Medical Centre, Sungkyunkwan University School of Medicine, Seoul, Korea; 3 Department of Anaesthesiology and Pain Medicine, CHA University Ilsan Medical Center, College of Medicine, CHA University of Korea, Seoul, Korea; Ohio State University Wexner Medical Center Department of Surgery, UNITED STATES

## Abstract

**Objectives:**

The occurrence of postoperative neurocognitive deficits(POND)after major cardiac surgery is associated with an increase in perioperative mortality and morbidity. Oxidative stress caused by oxygen can affect neuronal damage, which can lead to POND. Whether the intraoperative rSO_2_ value reflects oxidative stress and the associated incidence of POND is unknown.

**Methods:**

Among 3482 patients undergoing cardiac surgery, 976 patients were allocated for this retrospective study. Of these, 230 patients (32.5%) were observed to have postoperative neurologic symptoms. After propensity score 1:2 ratio matching, a total of 690 patients were included in the analysis. Recorded data on the occurrence of POND from the postoperative period to predischarge were collected from the electronic records.

**Results:**

The mean baseline rSO_2_ value was higher in the POND (–) group than in the POND (+) group. The mean overall minimum rSO_2_ value was lower in the POND (+) group (52.2 ± 8.3 vs 48.3 ± 10.5, *P* < 0.001). The mean overall maximum rSO_2_ values were not significantly different between the two groups (72.7 ± 8.3 vs 73.2 ± 9.2, *P* = 0.526). However, there was a greater increase in the overall maximum rSO_2_ values as compared with baseline in the POND (+) group (10.9 ± 8.2 vs 17.9 ± 10.2, *P* < 0.001). The degree of increase in the maximum rSO2 value was a risk factor affecting the occurrence of POND (adjusted odds ratio, 1.08; 95% confidence interval [CI], 1.04–1.11; P < 0.001). The areas under the receiver-operating characteristic curve for delta values of minimal and maximal compared with baseline values were 0.60 and 0.71, respectively.

**Conclusions:**

Increased cerebral oximeter levels during cardiac surgery may also be a risk factor for POND. This is considered to reflect the possibility of oxidative neuronal damage, and further studies are needed in the future.

## Introduction

The incidence of postoperative neurocognitive deficits (POND) after major cardiac surgery ranges from 25% to 80% [1,2]. Alterations in systemic and regional perfusion and oxygenation, exposure to anesthetic agents, cardiopulmonary bypass (CPB), and rapid changes in plasma pH and cellular metabolism during cardiac surgery induce oxidative stress that can contribute to postoperative delirium [3–5]. POND is associated with an increase in perioperative mortality and morbidity, longer hospital stay, and a prolonged rehabilitation process. Regional cerebral oxygen saturation (rSO2) is monitored using near-infrared spectroscopy (NIRS) during cardiac surgery to detect reduced oxygen supply to the brain [6,7].

The cerebral oximeter is widely used to detect changes in cerebral oxygen saturation and cerebral ischemia during cardiac surgery [8]. Previous studies have demonstrated that when brain oxygen saturation levels are reduced by more than 30% relative to baseline during cardiac surgery, a correlation is observed between POND, intensive care unit, and increased length of hospital stay [8–11]. However, these previous studies focused on the occurrence of neurocognitive deficits after surgeries in which the cerebral oximeter values decreased, and recent studies have focused on and proven that oxidative stress caused by oxygen can affect neuronal damage [4,5]. Lopez et al reported that oxidative damage is associated with occurrence of POND as a result of direct neuronal damage as well as destruction of the blood–brain barrier [12].

We hypothesized that not only the degree of decrease in rSO2 value but also the degree of increase in rSO2 value could affect the occurrence of POND. Therefore, we retrospectively investigated the association between the incidence of POND and intraoperative values of rSO2 in cardiac surgery.

## Method

### Patients

This retrospective study was approved by the institutional review board (IRB) of Samsung Medical Centre (approval No. 2019-02-083, March 5, 2019). Because of the retrospective nature of the study, in which the medical records of patients who had already undergone the operation were analyzed, the IRB waived the requirements for informed consent. The inclusion criterion was adult patients aged 20 years or older who underwent cardiac surgery on CPB between January 2015 and December 2018. For patients who underwent two or more surgeries, we included only the final surgery in the analysis. Exclusion criteria were pediatric patients, patients without a CPB period, and patients with missing data during surgery.

### Anesthesia and CPB

Anesthesia was induced, and total intravenous anesthesia was maintained with remifentanil, propofol, and rocuronium via peripheral line during the operation. The anesthetic agent was titrated for a bispectral index between 40 and 60. The maintenance fluid was crystalloid solution, infused at a rate of 5 mL∙ kg^−1^·h^−1^. Hemoglobin level was maintained at ≥ 9g/dL [13]. The rates of fluid administration and red blood cell transfusion were adjusted in consideration of estimated blood loss according to our institution’s protocol. Mechanical ventilation was controlled to maintain normocarbia (PaCO_2_ 4.7–5.0 kPa) throughout surgery, except during CPB, when a continuous positive airway pressure of 5 cmH_2_O with 50% oxygen was applied. Vasoactive drugs (dopamine, dobutamine, nitroglycerin, norepinephrine, milrinone, and epinephrine) were administered to maintain hemodynamic stability throughout the operation period. CPB was performed using bicaval cannulation with mild hypothermia. For myocardial protection, both intermittent cold blood cardioplegia via the antegrade or retrograde route and topical cooling with ice slush were used. In patients with significant aortic regurgitation, retrograde cardioplegia was infused using direct coronary sinus cannulation. PaCO_2_ and pH were managed during the CPB period in accordance with the a-stat strategy.

### Regional cerebral oximetry monitoring

Regional cerebral oximetry was obtained with NIRS monitoring performed using sensors placed bilaterally on the patient’s forehead during cardiac surgery (sensor and monitor: Medtronic/Covidien INVOS cerebral/somatic oximetry adult sensors, Somanetics Corporation, Troy, MI, USA). The baseline rSO_2_ value was obtained while the patient was breathing room air, and subsequent rSO_2_ values were recorded at 5-minute intervals throughout the duration of the surgical period, beginning 1 minute after the sensor was placed.

### Propensity score matching

All patients were divided according to the presence or absence of postoperative neurologic symptoms. To compensate for demographic imbalance, we performed propensity score matching between the two groups. This method consisted of determining cases and controls and then selecting the first case and finding the control with the closest propensity score. A logistic regression model was built given the covariates of age, sex, body mass index, presence of cerebrovascular accident history, type of operation, and comorbidities (e.g., diabetes mellitus, hypertension, arterial fibrillation, and myocardial infarction). We applied 1:2 nearest-neighbor matching without replacement to ensure that conditional bias was minimized. Propensity score matching was performed using the Matchlt package R (R Foundation for Statistical Computing).

#### Assessment of neurologic problem and data acquisition

We collected recorded data on the new development of POND from the postoperative period to predischarge from the electronic records. Diagnosed POND categorized as stroke, intracranial hemorrhage, delirium, seizure and coma. Unspecified PONDs and behaviors include cases such as agitation, confusion and delayed mental recovery, which were recorded in the electronic medical record but not registered as a diagnosis. (S1 Table) [14,15] Delirium assessment was performed using the confusion assessment method for the intensive care unit (CAM-ICU) [16]. When patients were discharged from the ICU, we assessed delirium using the confusion assessment method (CAM). Delirium, as defined by the CAM, was classified and categorized into four categories: acute onset of changes or fluctuations in the course of mental status, inattention, disorganized thinking, and altered level of consciousness. The CAM-ICU measures the acute onset or fluctuation of mental status changes, and patients were followed up daily with the Glasgow Coma Scale and an agitation/sedation scale (Richmond Agitation Sedation Scale; supplementary file) [17,18]. The CAM-ICU and CAM testers were assessed and recorded by individuals who received training and had been performing these tests for a number of years. Patients were assessed for delirium at least once daily until discharge, and the results were recorded in the electronic medical record. The primary outcome is the comparison of the predicted degree of POND occurrence according to the change in rSO_2_ value. The secondary outcome is the effect of changes at each stage of surgery on the occurrence of POND as compared with the baseline.

### Statistical analysis

We performed 1:2 propensity score matching, which is a method used to reduce confounding effects in observational studies [19]. Continuous variables were compared using the *t* test or Mann–Whitney *U* test as appropriate. Categorical variables were analyzed using Pearson’s chi-square test or Fisher’s exact test as appropriate. Data are presented as means (standard deviations). We performed logistic regression analysis to obtain the crude odds ratio and adjusted odds ratio of the values for each operation period’s rSO_2_ on POND. A receiver-operating characteristic (ROC) curve was constructed using cerebral oximeter values, and we calculated the area under the ROC (AUROC) to assess the prediction of POND. Cutoff values were set using the maximum of the Youden index (J). The Delong test was used to compare the AUROC between ROC curves. Statistical analyses were performed using SPSS version 22 (SPSS Inc.), R statistical software version 3.5.3 (Vienna, Austria; https://www.r-project.org/), and Medcalc software (Ostend, Belgium). A *P* value less than 0.05 was considered statistically significant.

## Results

**Fig 1:**
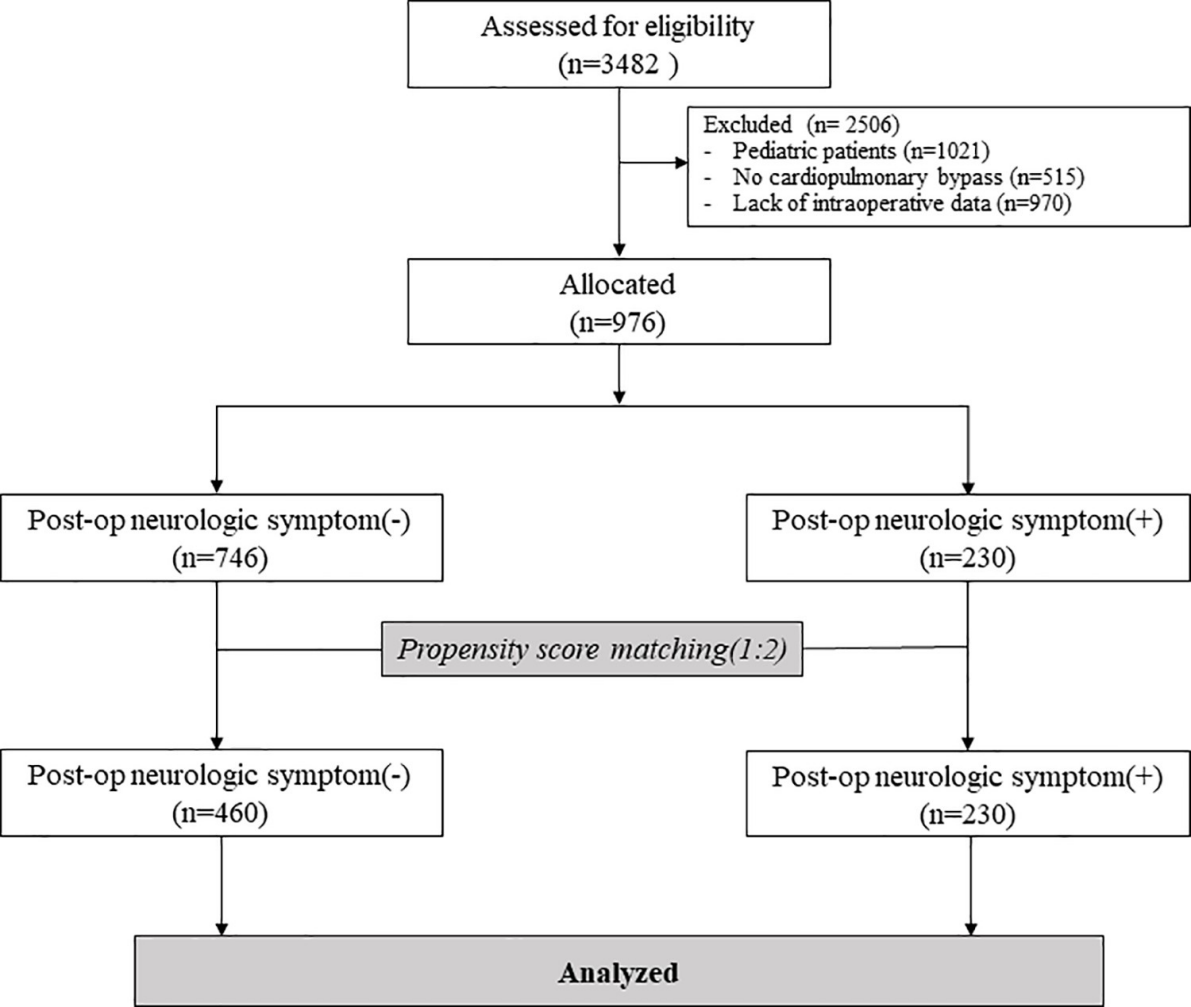
Flow diagram.

We assessed the study eligibility of 3482 patients (Fig 1). Of these, 2506 patients were excluded for the following reasons: (1) 1021 patients were pediatric patients, (2) 515 patients underwent cardiac surgery without CPB, and (3) 970 patients were excluded due to a lack of intraoperative cerebral oximeter data. Therefore, 976 patients were allocated for this retrospective study. Among these, 230 patients (32.5%) were observed to have postoperative neurologic symptoms. After propensity score 1:2 ratio matching, a total of 690 patients were included in the final analysis (Fig 1).

Table 1 shows the patient characteristics. Table 2 shows the rSO_2_ values for each operation period. In raw rSO2 values, baseline and overall minimum values showed significant differences between the two groups.(P<0.001, respectively) The baseline rSO_2_ value was lower in the POND (+) group than in the POND (-) group (61.9 ± 9.6 vs 55.3 ± 11.4; *P* < 0.001). The mean overall minimum rSO_2_ value was lower in the POND (+) group (52.2 ± 8.3 vs 48.3 ± 10.5, *P* < 0.001). There were significant differences between the two groups in changes of preweaning, postweaning, overall minimum, and overall maximum values compared to baseline(**Δ)**. In the POND (+) group, there was a significant increase in rSO2 values in the pre- and post-CPB weaning periods.(Δ(baseline–preweaning), 2.0 ± 10.1 vs 9.2 ± 13.1, P < 0.001; Δ(baseline–postweaning), 7.1 ± 9.6 vs 13.1 ± 11.4, P < 0.001). As compared with baseline, there was a lesser decrease in the overall minimum rSO_2_ values in the POND (+) group (−9.8 ± 7.7 vs −7.1 ± 8.7, P < 0.001). Overall maximal rSO2 values were greater increased compared to baseline in the POND(+) group (10.9 ± 8.2 vs 17.9 ± 10.2, P < 0.001).

**Table 1:** Baseline patient characteristics.

	Postoperative neurologic symptom (–) (n = 460)	Postoperative neurologic symptom (+) (n = 230)	*P* value
Gender (female/male)	224/236	111/119	0.979
Age, years	62.0 ± 14.6	62.6 ± 13.7	0.568
Weight, kg	60.2 ± 11.9	60.9 ± 11.8	0.473
Height, cm	161.2 ± 10.4	162.7 ± 10.0	0.074
BMI	23.1 ± 3.4	23.0 ± 3.6	0.736
CVA history, n (%)	106 (23)	70 (30)	0.045
Type of surgery, n(%)			
Valve	205(45)	108(47)	0.772
Aorta	125(27)	62(27)	
CABG	4(1)	2(1)	
Others*	126(27)	58(25)	
Baseline ABGA			
FiO_2_	0.6 ± 2.3	0.5 ± 0.2	0.345
PaCO_2_	35.9 ± 7.4	35.2 ± 4.7	0.176
PaO_2_	191.0 ± 87.2	196.0 ± 98.4	0.515
Glucose	116.7 ± 37.3	117.6 ± 36.7	0.775
Baseline MAP	72.1 ± 15.2	70.6 ± 13.7	0.189
Baseline CVP	7.1 ± 9.8	8.2 ± 6.6	0.080

BMI, body mass index; CVA, cerebrovascular accident; CVP, central venous pressure; MAP, mean arterial pressure; CABG, coronary artery bypass grafting.

* Type of others surgery included septal myectomy, VSD/ASD closure and cardiac mass removal.

Data are presented as the number of patients with percentage (%) or the mean ± SD.

**Table 2:** rSO_2_ values during the operation period.

rSO_2_ value	Postoperative neurologic symptom (–) (n = 460)	Postoperative neurologic symptom (+) (n = 230)	*P* value
**Baseline**			
Right	62.0 ± 10.2	54.8 ± 11.9	<0.001
Left	61.7 ± 9.8	55.7 ± 11.5	<0.001
Mean	61.9 ± 9.6	55.3 ± 11.4	<0.001
**Preweaning period**			
Right	64.0 ± 10.4	64.4 ± 12.2	0.674
Left	63.8 ± 10.0	64.6 ± 11.8	0.356
Mean	63.98 ± 9.8	64.58 ± 11.4	0.484
**Δ(baseline–preweaning)**			
Right	2.0 ± 10.6	9.5 ± 13.7	<0.001
Left	2.1 ± 10.4	8.9 ± 13.5	<0.001
Mean	2.0 ± 10.1	9.2 ± 13.1	<0.001
**Postweaning period**			
Right	68.9 ± 6.5	68.0 ± 11.0	0.279
Left	68.8 ± 9.5	68.6 ± 11.1	0.805
Mean	68.98 ± 9.0	68.38 ± 10.3	0.478
**Δ(baseline–postweaning)**			
Right	6.9 ± 9.9	13.2 ± 11.7	<0.001
Left	7.1 ± 10.0	12.9 ± 12.2	<0.001
Mean	7.1 ± 9.6	13.1 ± 11.4	<0.001
**Overall minimum**			
Right	52.1 ± 8.7	47.8 ± 11.5	<0.001
Left	52.1 ± 9.0	48.8 ± 10.4	<0.001
Mean	52.2 ± 8.3	48.3 ± 10.5	<0.001
**Δ(baseline–minimum)**			
Right	−10.0 ± 8.4	−7.1 ± 9.4	<0.001
Left	−9.4 ± 8.1	−6.9 ± 8.7	<0.001
Mean	−9.8 ± 7.7	−7.1 ± 8.7	<0.001
**Overall maximum**			
Right	73.0 ± 8.9	72.8 ± 9.8	0.828
Left	72.5 ± 8.6	73.6 ± 9.7	0.156
Mean	72.7 ± 8.3	73.2 ± 9.2	0.526
**Δ(baseline–maximum)**			
Right	11.0 ± 8.5	18.0 ± 10.6	<0.001
Left	10.8 ± 8.8	17.8 ± 10.7	<0.001
Mean	10.9 ± 8.2	17.9 ± 10.2	<0.001

Data are presented as the mean ± SD.

Table 3 shows the results of the multivariate logistic regression analysis on the variables that were significant in Table 2. The increase in the maximum rSO2 value compared to the baseline was a risk factor affecting the occurrence of POND (adjusted odds ratio, 1.08; 95% confidence interval [CI], 1.04–1.11; *P* < 0.001).

**Table 3:** Logistic regression analysis.

	Crude OR (95%CI)	Crude *P* value	Adjusted OR (95%CI)	Adjusted *P* value
**Baseline**	0.94 (0.92, 0.96)	<0.001	0.99 (0.96, 1.02)	0.415
**Δ(baseline–postweaning)**	1.05 (1.04, 1.07)	<0.001	1.02 (0.99, 1.04)	0.141
**Δ(baseline–postweaning)**	1.05 (1.03, 1.07)	<0.001	0.98 (0.95, 1)	0.084
**Overall minimum**	0.95 (0.94, 0.97)	<0.001	0.98 (0.96, 1.01)	0.164
**Δ(baseline–minimum)**	1.04 (1.02, 1.07)	<0.001	0.98 (0.96, 1.01)	0.175
**Δ(baseline–maximum)**	1.09 (1.07, 1.11)	<0.001	1.08 (1.04, 1.11)	<0.001

Data are presented as the mean (95% confidence interval).

Table 4 presents the perioperative variables. Duration of CPB, duration of aortic cross-clamp, anesthesia time, operation time, ventilation time, and mental recovery time were prolonged in the POND (+) group. Major organ mortality and morbidity were more frequent in the POND (+) group with new stroke, acute kidney injury, prolonged ventilation and death.

**Table 4:** Other variables.

	POND (–) (n = 460)	POND (+) (n = 230)	*P* value
^a^CPB duration, min	148.1 ± 63.3	181.1 ± 85.0	<0.001
^a^ACC duration, min	112.0 ± 54.4	135.2 ± 68.2	<0.001
^a^Anesthesia time, min	390.3 ± 104.0	448.4 ± 140.9	<0.001
^a^Operation time, min	318.2 ± 102.7	368.9 ± 140.0	<0.001
^b^Ventilation time, min	639.4 ± 1464.7	2278.0 ± 6428.0	<0.001
^b^Mental recovery, min	335.4 ± 1458.9	1518.8 ± 6437.6	0.006
^b^ICU stay, h	48.6 ± 96.9	109.1 ± 359.5	0.013
^b^ **MOMM**			
New stroke (%)	0(0.0)	36(15.7)	<0.001
AKI (%)	53(11.5)	57(24.8)	<0.001
Prolonged ventilation (%)	15(3.3)	48(20.9)	<0.001
Deep sternal infection (%)	9(2.0)	7(3.0)	0.531
Reoperation (%)	15(3.3)	12(5.2)	0.298
Death (%)	4(0.9)	22(9.6)	<0.001

Data are presented as the mean ± SD and percentage (%). ICU, intensive care unit; CPB, cardiopulmonary bypass; ACC, aortic cross-clamp; MOMM, major organ mortality; AKI, acute kidney injury.

^a^:CPB duration time, ACC duration time, Anesthesia time and Operation time are intraoperative data

^b^:Ventilation time, Mental recovery time, ICU stay and MOMM are postoperative data.

**Fig 2:**
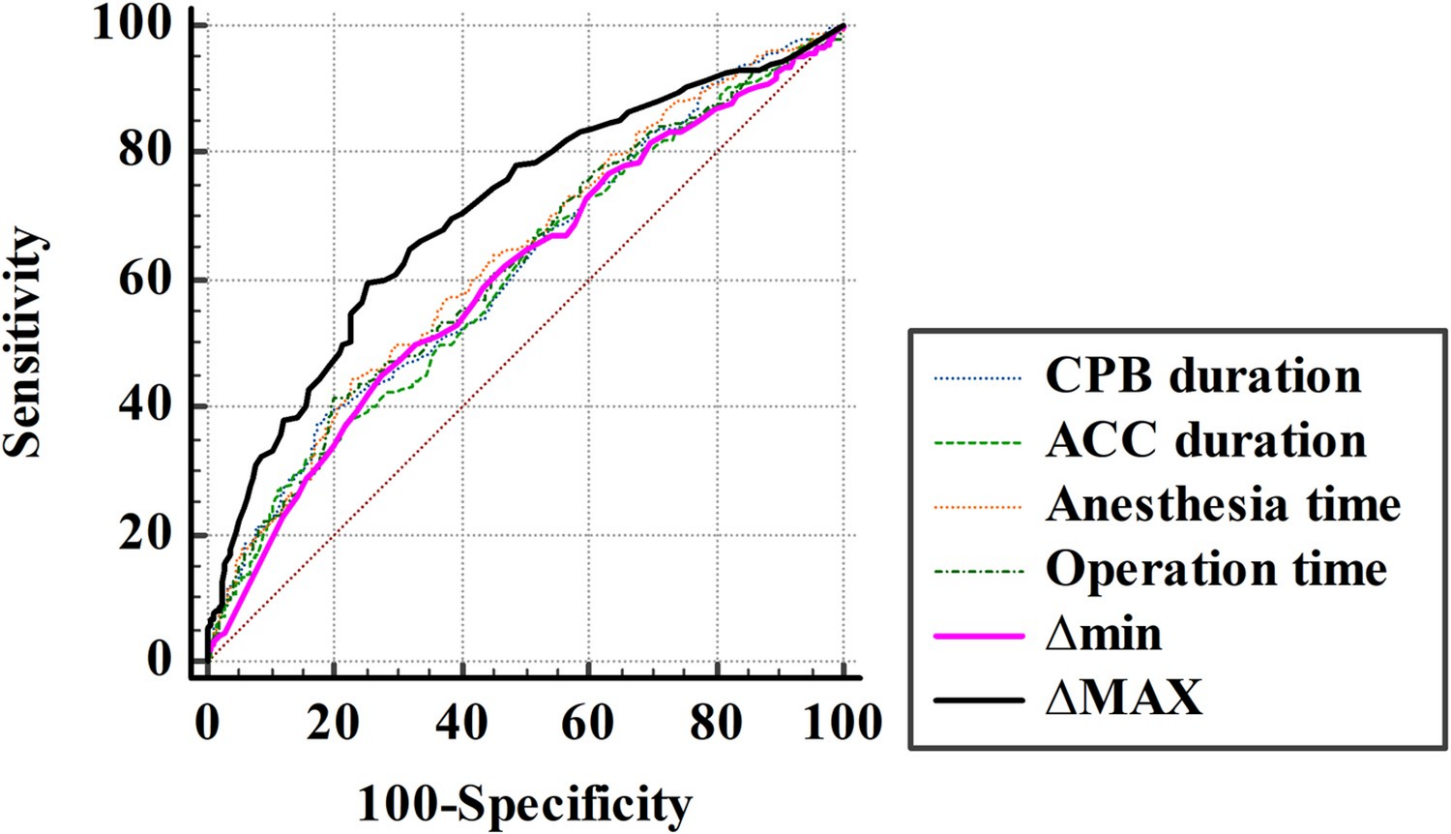
Receiver operating characteristics (ROC) curves. The AUROC for intraoperative data CPB duration, ACC duration, anesthesia time, operation time, Δmin and ΔMAX were 0.618, 0.603, 0.633, 0.619, 0.603 and 0.706, respectively. Δmin is Δ(baseline–maximum rSO_2_), and ΔMAX is Δ(baseline–minimum rSO_2_).

Fig 2 shows the ROC curve of intraoperative variables. The AUROC for delta values of minimal compared with baseline values(Δmin) was 0.60 (95% CI, 0.57–0.64). The AUROC for the delta values of maximal compared with baseline values(ΔMAX) was 0.71 (95% CI, 0.67–0.74). The delta values of maximal compared with baseline values were significantly more predictive of POND than the delta values of minimal compared with baseline values (*P* < 0.0001). The cut-off value of the delta maximal value was 15.5, and the sensitivity and specificity were 60% (95% CI, 53%–66%) and 75% (95% CI, 71%–79%), respectively. The cutoff value of the delta minimal value was −5, and the sensitivity and specificity were 44% (95% CI, 38%–52%) and 73% (95% CI, 68%–77%), respectively. The AUROC for intraoperative data CPB duration, ACC duration, anesthesia time, operation time, were 0.62(0.58–0.65), 0.60(0.57–0.64), 0.63(0.60–0.67) and 0.62(0.57–0.66), respectively. There was no statistically significant difference of AUROC between CPB duration, ACC duration, anesthesia time, operation time and Δmin. (P>0.05)

## Discussion

This retrospective study found that increased rSO2 values during operation may predict the development of POND. To the best of our knowledge, this is the first study to confirm retrospectively that not only a decrease in rSO_2_ but also an increase in rSO_2_ may affect the occurrence of POND. The use of cerebral oximeter is essential to predict the development of POND during major cardiac surgery.

The mechanism underlying the occurrence of POND in cardiac surgery remains unclear. Most previous studies focused on the desaturation event on rSO_2_, which could adversely affect the incidence of POND and other postoperative outcomes. It is known that when the cerebral oximetry value decreases by more than 30% as compared with baseline, it is defined as cerebral desaturation [10]. Among patients aged 70 years or older who underwent coronary artery bypass graft surgery, those with a reduction of ≥50% compared with baseline rSO_2_ had a high incidence of early-onset POND, and those with a reduction of ≥30% compared with baseline rSO_2_ had a high incidence of late-onset POND [20]. In contrast, the findings of a recent prospective randomized trial reported no difference between intraoperative rSO_2_ values and POND after cardiac surgery [21]. That is, the decrease in rSO_2_ was not absolutely predictive of POND. The research trend on the relationship between rSO_2_ and the occurrence of POND has been changing.

Meanwhile, there is no controversy about the possibility of organ injury after the transition from the ischemic state to hyperoxic reperfusion during surgery [22]. There is a possibility of hyperoxic damage to brain tissue during reperfusion and during oxygenation after the transition from CPB to spontaneous circulation during cardiac surgery [23]. Hyperoxic reperfusion-induced vasoconstriction or oxidative injury are mechanisms for the association between hyperoxic cerebral reperfusion and POND [24,25]. During hyperoxic reperfusion, there is an increase in oxidative neuronal damage, and F2-isoprostanes and isofurans increase in plasma as markers of systemic oxidative neuronal damage [26,27]. F2-isoprostanes are associated with brain arteriole vasoconstriction, and isofurans have been found to be mediators between hyperoxia and POND [28,29]. In addition to F2-isoprostanes and isofuran, S100 calcium-binding protein B, an indicator of disruption to the blood–brain barrier by oxidative injury, was also higher in the POND group during cardiac surgery [5]. That is, the possibility that the blood–brain barrier can be destroyed by systemic oxidative damage during surgery and cause POND by neuronal injury was proven.

On the other hand, the association between the occurrence of POND and an increase in rSO_2_ cannot be ruled out due to cerebral bleeding. CPB reduces platelet counts by about 50% and causes platelet dysfunction as well as reduced levels of clotting factors and von Willebrand factors [30,31]. A large volume of heparin is administered during CPB, and in the CPB-weaning process, it reverses to protamine. This process may have a rebound effect of heparin, which causes bleeding after CPB weaning.^26^ In a study of pediatric patients to detect intracerebral hemorrhage (ICH) using NIRS, 21 patients showed a ≥45% increase in NIRS value, 12 patients of whom had an actual ICH (sensitivity = 100%, specificity = 80%) [32]. When acute hemorrhage occurs, hemoglobin aggregation temporarily increases the cerebral oximeter level. In our study, we observed ICH in nine patients, and the maximal cerebral oximeter value increased by an average of 47% as compared with baseline. The rapid increase in rSO_2_ during conversion from CPB to spontaneous flow also indicates that the possibility of ICH cannot be excluded. However, an increase in the cerebral oximeter value is not an absolute marker of ICH during cardiac surgery, because of the effects of anesthesia, ventilator, CPB flow, and so forth. Thus, there is a need for further prospective studies in the future.

Unfortunately, there is no gold standard to establish the diagnosis of POND. Therefore, the incidence of POND/POCD is dependent on definitions based on in each study. We applied the concept of postoperative neurocognitive deficits (POND), which combines the concepts of postoperative cognitive dysfunction (POCD) and postoperative delirium(POD). In a retrospective study, there is a limit to clearly distinguishing POCD from POD only with electronic medical records. In addition, previous studies demonstrated that POD and POCD occasionally occur in the same individual with overlapping risk factors have suggested a common underlying neuropathogenesis [15,33].

There are several limitations to our study. First, Study results may be affect confounding factors such as postoperative anxiety, pain and medications. The occurrence of POND/POCD is strongly dependent on expert opinion. Therefore, there is a possibility of false positives if the observer does not distinguish the confounding factors. Second, we cannot confirm the specific time point exactly. Most of the preweaning periods and postweaning periods in which most cerebral oximeter values were increased during surgery had maximal cerebral oximeter values, but the exact time point could not be determined. Third, we did not measure the O2 concentration in the actual cerebral blood flow, It cannot not be sure that an increase in rSO2 is real hyperoxia. Our study was limited in confirming the exact correlation between cerebral oximeter and PaO2 as a retrospective study. However, our results suggest that the possibility of neuronal damage due to increased oxygenation cannot be completely ruled out

In conclusion, excessively increased cerebral oximeter values during major cardiac surgery may predict the development of POND. The increased cerebral oximeter value is considered to reflect the possibility of oxidative neuronal damage, and further studies are needed in the future.

## Supporting information

S1 Table Classification of POND. (DOCX)
