# Supplementary material for: Effect of changes in cerebral oximeter values during cardiac surgery on the incidence of postoperative neurocognitive deficits (POND): A retrospective study based on propensity score–matched analysis
Source: PLoS One. 2021 Dec 3;16(12):e0260945. doi: 10.1371/journal.pone.0260945 (PMC8641887; doi:10.1371/journal.pone.0260945)
Supplement: S1 Table — (DOCX) [file pone.0260945.s001.docx]

**Supplementary table 1. Classification of POND.**

|  | **N(%)** |
| --- | --- |
| **Diagnosed POND** | **126(53.9)** |
| Stroke | 27(11.7) |
| ICH | 20(8.7) |
| Delirium* | 47(20.4) |
| Seizure | 27(11.7) |
| Coma | 3(1.3) |
| **Unspecified PONDs and behaviors** | **104(46.1)** |

**Data are presented as number(percentage).**

**POND, postoperative neurocognitive deficits; ICH, intracranial hemorrhage;**

***Delirium was diagnosed by physicians.**
